# Social vulnerability and severe COVID-19 in pregnant women: an ecological study in Pernambuco State, Brazil, 2020-2021

**DOI:** 10.1590/0102-311XEN175623

**Published:** 2025-03-31

**Authors:** Hingrid Wandille Barros da Silva Sá, Mirella Bezerra Rodrigues Vilela, Carlos Fabrício Assunção da Silva, Gabriella Morais Duarte Miranda, Heitor Victor Veiga da Costa, Cristine Vieira do Bonfim

**Affiliations:** 1 Universidade Federal de Pernambuco, Recife, Brasil.; 2 Instituto Aggeu Magalhães, Fundação Oswaldo Cruz, Recife, Brasil.; 3 Fundação Joaquim Nabuco, Recife, Brasil.

**Keywords:** Pregnant Women, COVID-19, Social Vulnerability, Mujeres Embarazadas, COVID-19, Vulnerabilidad Social

## Abstract

This study analyzed the association between social vulnerability indicators and the incidence rate of severe COVID-19 in pregnant women in Pernambuco State, Brazil, between 2020 and 2021. It is an ecological study that assessed severe cases of COVID-19 in pregnant women reported to the Influenza Surveillance System in Brazil. To determine such association, the zero adjusted Gamma (ZAGA) regression model was applied due to the large number of zeros in the response variable. Variables available in the Demographic Census were used, representing socioenvironmental conditions, household characteristics, and urban services. In the study period, 475 severe cases of COVID-19 were reported in pregnant women, with an incidence rate of 1.40 cases per 1,000 live births. Modeling with ZAGA showed that the mean incidence rate is affected by the illiteracy rate, with the average increasing by a relative 5.1% for every 1% (p = 0.024). The ZAGA model also estimates the chance of a municipality having a zero rate, with these values increasing by 2.7% for every 1% of the proportion of Family Health Strategy coverage, by 19.3% for every 0.01 of the Municipal Human Development Index (M-HDI) education dimension, and by 21.3% for every 0.01 of the M-HDI longevity dimension. When the M-HDI increases, the chance of the municipality having a zero rate decreases by 33.8% for every 0.01. Population density reduces the chance by 4.5% for every 10 inhabitants/km^2^. This study highlighted the influence of social vulnerability indicators on the incidence of severe COVID-19 cases in pregnant women, showing that some aspects of social and demographic characteristics are related to such influence.

## Abstract (es)

Se analizó la asociación de indicadores de vulnerabilidad social con la tasa de incidencia de casos graves del COVID-19 en mujeres embarazadas en Pernambuco, Brasil, entre 2020 y 2021. Se trata de un estudio ecológico con los casos graves de mujeres embarazadas con COVID-19 reportados en el Sistema de Información de Vigilancia Epidemiológica de Influenza. Para la asociación entre la vulnerabilidad social y estos casos, se aplicó la distribución gamma ajustada a cero (ZAGA), debido a la gran cantidad de ceros en la variable de respuesta. Se utilizaron variables disponibles en el censo demográfico que representan condiciones socioambientales, características del hogar y servicios urbanos. Se encontraron 475 casos graves del covid-19 en mujeres embarazadas, con una tasa de incidencia de 1,40 por 1.000 nacidos vivos. El modelo ZAGA mostró que la tasa promedio de incidencia de casos se ve afectada por la tasa de analfabetismo, por lo cual el promedio aumenta relativamente un 5,1% (p = 0,024) por cada 1%. Este modelo también estima la probabilidad de que un municipio tenga una tasa cero, y estos valores aumentan en un 2,7% por cada 1% de proporción de cobertura de la Estrategia de Salud Familiar, en un 19,3% por cada 0,01 puntos de nivel de estudios en el Índice Municipal de Desarrollo Humano (IDH-M) y en un 21,3% por cada 0,01 puntos de longevidad en el IDH-M. Cuando aumenta el IDH-M, la probabilidad de que el municipio tenga una tasa cero disminuye en un 33,8% por cada 0,01 puntos. La densidad demográfica disminuye la probabilidad en un 4,5% por cada 10 habitantes/km^2^. Este estudio mostró la influencia de los indicadores de vulnerabilidad social en la incidencia de casos graves del COVID-19 en mujeres embarazadas, los cuales se relacionan con algunos aspectos como las características sociales y demográficas.

## Introduction

The pandemic of COVID-19 has alarmed the whole world and has promoted debates on the measures to be taken against the disease. These strategies are linked to the evidence that some groups are at greater risk of complications from COVID-19 ^1^. Physiological changes during pregnancy predispose pregnant women to more severe forms of COVID-19 ^2^ ^,^ ^3^; therefore pregnant women represent a risk group for infection and a priority for care and testing ^2^.

The COVID-19 virus spread rapidly throughout the world, causing social, economic, and health problems in several countries ^4^. A study conducted in Brazil ^5^ assessed the relationship between the incidence, mortality, and fatality rates of COVID-19 and social indicators of human development and social vulnerability. The study found that there are concerns about the impact of the COVID-19 pandemic on the poorest populations, particularly in low- and middle-income countries, due to barriers to adoption of preventive measures. These groups are exposed to vulnerabilities that increase the risk of contamination, with limited access to health services if infection occurs.

As with other health problems, social vulnerability can influence the risk of infection, morbidity, and mortality associated with COVID-19 ^1^. Social vulnerability has a multidimensional definition and affects individuals or groups face in situations of fragility due to biological, epidemiological, social, and/or cultural factors, exposing them to risks and significant levels of social disintegration ^6^.

Several scientific studies have highlighted the social inequalities resulting from the COVID-19 pandemic ^7^ ^,^ ^8^. These studies revealed racial and ethnic ^9^ ^,^ ^10^, socioeconomic ^11^, and territorial inequalities in mortality associated with COVID-19 ^12^. Together with preexisting social inequalities, the COVID-19 pandemic has further emphasized the global issue of social inequities in health ^13^.

Health vulnerability mainly affects people in less favorable socioeconomic situations, with limited access to health care and poor quality services ^14^. Assessments of the association of social vulnerability with severe COVID-19 in pregnant women can support the implementation of measures to control the spread of the virus and the planning of resource allocation. Based on this perspective, this study aimed to analyze the association of social vulnerability indicators with the incidence rate of severe COVID-19 cases in pregnant women in Pernambuco State, Brazil between 2020 and 2021.

## Materials and methods

### Study location

This study used an ecological approach, with municipalities in the state of Pernambuco as the unit of analysis. The state of Pernambuco, located in the Northeast region of Brazil, has 184 municipalities, in addition to the Fernando de Noronha Archipelago. According to the 2022 *Demographic Census*, the total population of the state was 9,058,931.

### Data source

The study included all confirmed cases of pregnant women with COVID-19 and clinical manifestations classified by the Ministry of Health as severe, reported from March 2020 to December 2021 in Pernambuco State. Severe cases were extracted from the Influenza Epidemiological Surveillance Information System (SIVEP-Gripe, acronym in Portuguese), a national database created by the Brazilian Ministry of Health in 2009 for influenza syndrome surveillance and which included the notification of SARS cases and deaths caused by SARS-Cov-2. The variable observed in SIVEP-Gripe for spatial analysis was the municipality of residence. The e-SUS Notifica was used to obtain the number of COVID-19 cases in pregnant women during the study period.

The variables used to extract data from the severe acute respiratory syndrome (SARS) hospitalized patients database were: date of form completion (between March 2020 and December 2021); municipality of notification (municipalities in the state of Pernambuco); sex (female); pregnant woman (gestational age of the patient). The exclusion criterion was maternal age under 11 years and over 60 years. SARS cases were considered as the presentation of dyspnea/respiratory distress or persistent pressure in the chest or oxygen saturation below 95% in room air or bluish lips or face ^15^.

Social, demographic, and economic data extracted from the 2010 demographic census were used to analyze the association with social vulnerability. For the proportion of Family Health Strategy (FHS) coverage, 2020 data from the e-Gestor Primary Health Care system (https://egestoraps.saude.gov.br/) were used. This system was developed by the Brazilian Ministry of Health to offer information about primary care systems.

The indicators used to analyze social vulnerability were: Social Vulnerability Index (SVI), Municipal Human Development Index (M-HDI), education Index of the Municipal Human Development Index (education M-HDI), longevity Index of the Municipal Human Development Index (longevity M-HDI), Income Index of the Municipal Human Development Index (income M-HDI), the Gini Index, unemployment rate, per capita income, illiteracy rate for people aged 15 and over, proportion of the population with inadequate sewage, proportion of the population with homes with tap water, proportion of adequate garbage collection, proportion of the population with adequate bathrooms and water supply, population with homes with garbage collection, proportion of the population with homes with electricity, urbanization rate, and population density.

To calculate the incidence rate of severe cases in pregnant women, the number of live births in 2020 and 2021 obtained from the Brazilian Information System on Live Birth (SINASC, acronym in Portuguese) was used in the denominator.

### Data analysis

To determine the influence of social vulnerability indicators on the incidence rate of severe cases of COVID-19 in pregnant women per 1,000 live births (response variable), the rate was calculated for each municipality in Pernambuco for the period of 2020 to 2021 (where the average between the two years was used), taking into account the municipality of residence of the pregnant woman, using the following equation:

$${Rate}_{i}=\frac{TotalnumberofseverecasesofCOVID-19inpregnantwomen}{Livebirths_{i}}1,000$$

in which *i* = municipality considered in the calculation.

Because the response variable presented a high number of zeros, it was difficult to model it with usual regression models. Then, for the modeling stage, two zero-adjusted probabilistic models were tested. The first was the zero adjustment Gamma (ZAGA) model and the second was the zero adjustment inverse Gaussian (ZAIG) model, using the generalized Akaike information criteria (GAIC). These models were adopted because the response variable is a rate, which presents values strictly greater than or equal to zero, and these models are adequate for modeling this type of variable, since it is supported in this range. For model selection, the lowest GAIC value was used [ZAGA (GAIC = 193.1798); ZAIG (GAIC = 199.9904)]; therefore, the ZAGA model was selected. The GAIC at this stage was calculated considering an adjustment of the probability density function of the proposed models to the histogram of the response variable data.

These models have three parameters in their probability distributions, which can be modeled simultaneously using the Generalized Additive Model for Location, Scale and Shape (GAMLSS) framework ^16^. The first parameter refers to the mean (*µ*), the second is about its variation (*σ*), and the third estimates the probability of a municipality having a rate equal to zero (*ν*). For each parameter, the statistical model was subjected to the variable selection process using the stepwise algorithm, using GAIC as a metric ^17^.

The functional form to be defined for the ZAGA model is presented as follows:

*Y~ZAGA(μ,σ,ν)*

*log(μ)=β_0+β_1 X_1+β_2 X_2+...+β_p X_p*

*log(σ)=α_0+α_1 X_1+α_2 X_2+...+α_p X_p*

*logit(ν)=γ_0+γ_1 X_1+γ_2 X_2+...+γ_p X_p*

in which *log*(.) is the Napierian logarithm, $logit(x)=\frac{1}{1+e^{-x}}$ is the logistic function, and *p =* number of explanatory variables.

Therefore, each parameter will have a different interpretation. For parameters that have a *log*(.) link function (e.g. *μ*, the mean), the relationship of interest can be evaluated by applying *exp*(*β*_*i*_ *u) to the parameter *β*_*i*_ of the variable of interest, with an interpretation for each unit of measurement u of variable *X*_*i*_ under evaluation. The parameter will increase/decrease on a percentage scale when compared to lagged values of *X*_*i*_ .

For the parameter that has a *logit*(.) link function, the same rule applies to the others; however, its interpretation is that the chance of the rate having a value of zero will increase/decrease on a percentage scale when compared to lagged values of *X*_*i*_ .

A bivariate analysis was performed to select the variables of the statistical model, using two different methods. The first method considered only municipalities with non-zero rates (for subsequent inclusion in the variable selection method for the *μ* parameter), while the second method investigated the probability of a municipality having a zero rate, considering all municipalities (for subsequent inclusion in the variable selection method for the *ν* parameter). Spearman’s correlation coefficient was used in the first method, while the Mann-Whitney test was used in the second. All variables that presented p < 0.20 in the bivariate analysis were included in the variable selection process via stepwise method. The value of p = 0.20 was adopted to be more permissive when testing the inclusion of variables in the statistical model. It should be noted that modeling could have used discrete distributions to model counting processes such as Poisson or negative binomial distribution using the population at risk as an offset. However, these models are much more computationally complex to estimate (when compared to models that use continuous distributions), which makes its use more difficult.

Spearman’s correlation measures the strength of the relationship between two variables in a non-linear way (non-increasing/decreasing monotonic relationships). The Mann-Whitney test is used to compare, in a non-parametric way, the difference between the distribution of two populations which, in this case, are the municipalities with and without rates of severe cases equal to zero.

Regarding the variable selection stage, the calculation of correlations is a screening stage for the stepwise variable selection algorithm. Therefore, we did not select the variables based on the correlations, but rather through the variable selection algorithm, which is a much more robust and judicious method.

Regarding the correlation between the M-HDI education and the illiteracy rate, they are in fact correlated. However, they are allocated to different parameters of the distribution, and the worm plot analysis did not present any problems, which indicates that the inclusion of two variables in the model does not affect the estimation of their parameters nor the adjustment quality.

To ensure the quality of findings from the regression analysis, a residual analysis was performed with the following evaluations: normality of residuals using the Shapiro-Wilk, Jarque-Bera, Kolmogorov-Smirnov, Anderson-Darling, Cramer-von Mises, Lilliefors, Pearson, and Shapiro-Francia tests; stationarity of residuals using the augmented Dickey-Fuller, Philips-Perron, and KPSS (Kwiatkowski-Phillips-Schmidt-Shin) tests (plateau and trend); autocorrelation of residuals using the Box-Pierce and Ljung-Box tests. Plots of residuals and adjusted values and time were also evaluated, as well as a density plot for residuals and a qq-plot considering a standard normal distribution.

The framework used to estimate the models (GAMLSS − https://www.gamlss.com/) has a graphical representation for the residuals named worm plot, which helps identify potential factors for the poor adjustment of a model. This plot was also used to complete the residual analysis.

All calculations related to the regression were performed using R version 4.0.1 (http://www.r-project.org) with the help of package GAMLSS version 5.4-3 ^18^. The significance level was 5% (for interpretation of the statistical significance of the model parameters after variable selection).

This research project had the consent of the State Health Department of Pernambuco for the access to the database and was approved by the Research Ethics Committee of Federal University of Pernambuco (CAAE: 60548022.1.0000.5208; report n. 5.544.400).

## Results

A total of 4,121 cases of COVID-19 in pregnant women were reported to e-SUS Notifica system in the study period, of which 475 (11.5%) were reported as severe COVID-19 in the SIVEP-Gripe system. Table 1 shows the descriptive statistics related to social vulnerability indicators and the incidence rate of severe cases of COVID-19 in pregnant women. The average rate (± standard deviation) of all municipalities was 1.40 per 1,000 live births (±1.78) of severe COVID-19 in pregnant women. The standard deviation was much higher than the average due to the number of municipalities with a zero rate, which corresponds to 46.2% (85) of the municipalities in Pernambuco State.

**Table 1:** Descriptive statistics of social vulnerability indicators for the municipalities in Pernambuco State, Brazil, 2020 and 2021.

Parameter	Minimum	1st quartile	Median	Mean	3rd quartile	Maximum	SD	Coefficient of variation
Rate of pregnant women with severe COVID-19 *	0.00	0.00	0.84	1.41	2.23	8.98	1.78	1.27
M-HDI	0.49	0.57	0.59	0.60	0.61	0.77	0.05	0.08
SVI	0.31	0.43	0.48	0.47	0.51	0.66	0.07	0.14
Gini index	0.42	0.49	0.52	0.52	0.55	0.68	0.05	0.09
Unemployment rate	0.00	0.19	0.30	0.35	0.49	1.00	0.21	0.61
M-HDI education	0.35	0.44	0.49	0.49	0.52	0.70	0.07	0.13
M-HDI longevity	0.68	0.73	0.76	0.76	0.78	0.84	0.03	0.05
M-HDI income	0.48	0.54	0.57	0.57	0.60	0.80	0.04	0.08
Per capita income	155.49	234.88	271.18	295.96	329.66	1,144.26	104.49	0.35
Illiteracy rate (15 years and older)	0.06	0.23	0.27	0.27	0.32	0.43	0.07	0.26
Proportion of homes with inadequate sewage	0.00	0.11	0.23	0.25	0.35	0.75	0.16	0.62
Proportion of population with tap water	0.00	0.58	0.74	0.71	0.85	1.00	0.19	0.26
Proportion of population with adequate bathroom and water supply	0.00	0.45	0.62	0.60	0.79	1.00	0.23	0.38
Proportion of population with adequate garbage collection	0.00	0.69	0.84	0.78	0.92	1.00	0.19	0.24
Proportion of population with electricity	0.00	0.85	0.91	0.86	0.95	1.00	0.16	0.19
Urbanization rate	0.12	0.46	0.62	0.62	0.77	1.00	0.20	0.33
Population density	7.79	39.82	87.24	247.04	154.06	9,068.36	905.99	3.67
Proportion of FHS coverage	0.00	0.93	1.00	0.93	1.00	1.00	0.14	0.15

FHS: Family Health Strategy; M-HDI: Municipal Human Development Index; SD: standard deviation; SVI: Social Vulnerability Index.

* To calculate this rate, a total of 4,121 cases of COVID-19 were reported to e-SUS Notifica, of which 475 were pregnant women with severe COVID-19.

Table 2 shows the Spearman’s correlation between the variables related to social vulnerability indicators and the incidence rate of severe cases of COVID-19 in pregnant women. Of note, variables M-HDI, the Gini index, M-HDI education, illiteracy rate, proportion of the population with tap water, and the proportion of FHS coverage showed p-values less than or equal to 0.20.

**Table 2:** Spearman’s correlation between the explanatory variables and the response variable of the incidence rate of severe COVID-19 in pregnant women, not including those municipalities with a rate equal to zero. Pernambuco State, Brazil, 2020 and 2021.

Parameter	Correlation	p-value
M-HDI	-0.146	0.150
SVI	0.037	0.713
Gini index	-0.226	0.025
Unemployment rate	-0.121	0.235
M-HDI education	-0.133	0.188
M-HDI longevity	-0.087	0.390
M-HDI income	-0.129	0.202
Per capita income	-0.127	0.211
Illiteracy rate (15 years and older)	0.161	0.111
Proportion of population with inadequate sewage	0.116	0.254
Proportion of population with tap water	-0.136	0.180
Proportion of population with adequate bathroom and water supply	-0.106	0.298
Proportion of population with adequate garbage collection	-0.002	0.984
Proportion of population with electricity	0.099	0.330
Urbanization rate	-0.121	0.235
Population density	-0.019	0.855
Proportion of FHS coverage	0.220	0.028

FHS: Family Health Strategy; M-HDI: Municipal Human Development Index; SVI: Social Vulnerability Index.

Note: values in bold, significant tests at the 0.20 level.

Regarding the association between variables of social vulnerability indicators and whether a municipality has a zero rate or not (Table 3), it can be seen that the SVI, the Gini index, and the proportion of population with adequate garbage collection did not present statistical significance. The other variables were eligible for multivariate analysis, and municipalities with a zero rate tended to have lower central values (median/mean) for: M-HDI, unemployment rate, M-HDI education, M-HDI longevity, M-HDI Income, per capita income, proportion of population with tap water, proportion of population with adequate bathroom and water supply, proportion of population with electricity, and population density.

**Table 3:** Descriptive statistics of social vulnerability indicators for municipalities stratified according to zero rate of severe COVID-19 in pregnant women. Pernambuco State, Brazil, 2020-2021.

Parameter	Municipalities with a rate equal to zero	Minimum	1st quartile	Median	Mean	3rd quartile	Maximum	SD	Coefficient of variation	p-value *
M-HDI	No	0.52	0.57	0.60	0.61	0.64	0.77	0.05	0.09	0.00
	Yes	0.49	0.57	0.59	0.58	0.60	0.67	0.03	0.06	
SVI	No	0.31	0.42	0.47	0.46	0.51	0.66	0.07	0.16	0.08
	Yes	0.36	0.45	0.49	0.48	0.51	0.62	0.06	0.12	
Gini index	No	0.43	0.49	0.52	0.52	0.55	0.68	0.05	0.09	0.97
	Yes	0.42	0.49	0.52	0.52	0.55	0.63	0.04	0.08	
Unemployment rate	No	0.00	0.24	0.36	0.39	0.53	1.00	0.21	0.55	0.00
	Yes	0.01	0.16	0.25	0.31	0.41	0.87	0.20	0.66	
M-HDI education	No	0.37	0.45	0.50	0.50	0.55	0.70	0.07	0.14	0.02
	Yes	0.35	0.44	0.48	0.47	0.52	0.58	0.05	0.11	
M-HDI longevity	No	0.68	0.73	0.76	0.76	0.78	0.84	0.04	0.05	0.06
	Yes	0.68	0.73	0.75	0.75	0.77	0.81	0.03	0.04	
M-HDI income	No	0.50	0.55	0.58	0.59	0.61	0.80	0.05	0.09	0.00
	Yes	0.48	0.54	0.56	0.56	0.58	0.65	0.03	0.05	
Per capita income	No	175.50	249.20	292.70	323.90	363.60	1,144.30	128.10	0.40	0.00
	Yes	155.50	232.30	256.30	263.40	297.00	441.80	51.40	0.20	
Illiteracy rate (15 years and older)	No	0.06	0.21	0.26	0.25	0.32	0.40	0.08	0.31	0.00
	Yes	0.15	0.25	0.29	0.29	0.33	0.43	0.06	0.19	
Proportion of population with inadequate sewage	No	0.00	0.09	0.18	0.21	0.31	0.71	0.16	0.73	0.00
	Yes	0.05	0.19	0.30	0.30	0.40	0.75	0.14	0.49	
Proportion of population with tap water	No	0.22	0.65	0.81	0.75	0.90	1.00	0.18	0.24	0.00
	Yes	0.00	0.52	0.67	0.65	0.79	0.98	0.18	0.27	
Proportion of population with adequate bathroom and water supply	No	0.07	0.51	0.72	0.67	0.83	1.00	0.23	0.34	0.00
	Yes	0.00	0.40	0.52	0.53	0.68	0.99	0.21	0.40	
Proportion of population with adequate garbage collection	No	0.00	0.69	0.84	0.78	0.92	1.00	0.19	0.25	0.77
	Yes	0.03	0.69	0.85	0.79	0.91	1.00	0.19	0.24	
Proportion of population with electricity	No	0.00	0.89	0.93	0.89	0.97	1.00	0.16	0.18	0.00
	Yes	0.12	0.76	0.88	0.83	0.93	0.99	0.16	0.19	
Urbanization rate	No	0.06	0.21	0.26	0.25	0.32	0.40	0.08	0.31	0.00
	Yes	0.15	0.25	0.29	0.29	0.33	0.43	0.06	0.19	
Population density	No	8.00	58.10	118.80	390.00	196.40	9,068.40	1218.10	3.10	0.00
	Yes	7.80	27.50	58.30	80.60	104.90	328.20	71.40	0.90	
Proportion of FHS coverage	No	0.45	0.86	1.00	0.91	1.00	1.00	0.14	0.16	0.00
	Yes	0.00	1.00	1.00	0.97	1.00	1.00	0.12	0.13	

FHS: Family Health Strategy; M-HDI: Municipal Human Development Index; SVI: Social Vulnerability Index.

Note: values in bold, significant tests at the 0.05 level.

* Mann-Whitney test.

On the other hand, the following variables: illiteracy rate (15 years or older), proportion of population with inadequate sewage, and urbanization rate had higher central values for municipalities with a zero rate.

The proportion of FHS coverage had the same median for both strata of municipalities; however, its mean was higher in municipalities with a zero rate. It indicates that below the median, the coverage proportion of municipalities with a zero rate tends to be higher in relation to municipalities with a rate other than zero.

**Figure 1:**
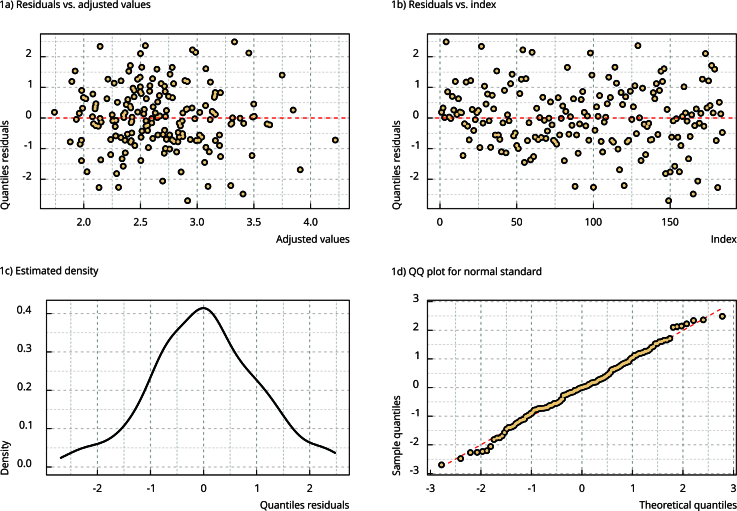
Graphs referring to residual analysis.

**Figure 2:**
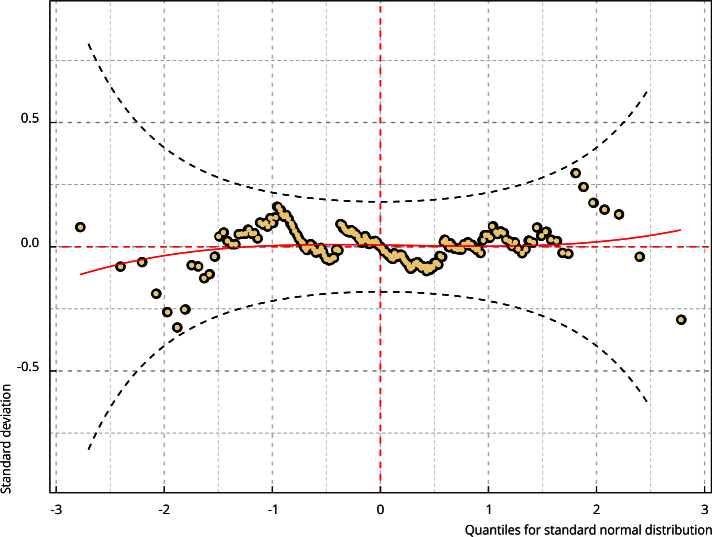
Worm plot showing model residuals.

The analysis of the residuals is presented in Figures 1 and 2 and Table 4. None of the tests showed nonconformities regarding the model residuals (normality, stationarity, and autocorrelation). Figure 1 also did not show any divergence regarding the assumptions of heteroscedasticity and others. Finally, the worm plot (Figure 2) highlights all points within the confidence bands, which is a sign of no significant divergences of the residuals. Therefore, the results of the regression model can be considered reliable.

**Table 4:** Statistical tests performed to assess normality, stationarity, and autocorrelation in the residuals of the zero-adjusted Gamma model (ZAGA).

Test performed	p-value	Result
Shapiro-Wilk	0.51	It is normal
Jarque-Bera	0.98	It is normal
Kolmogorov-Smirnov	0.82	It is normal
Anderson-Darlin	0.45	It is normal
Cramer-von Mises	0.41	It is normal
Lilliefors	0.37	It is normal
Pearson	0.65	It is normal
Shapiro-Francia	0.59	It is normal
Augmented Dickey-Fuller	0.01	It is stationary
Philips-Perron	0.01	It is stationary
KPSS for Level	0.10	It is stationary
KPSS for Trend	0.10	It is stationary
Box-Pierce	1.00	No autocorrelation
Ljung-Box	1.00	No autocorrelation

KPSS: Kwiatkowski-Phillips-Schmidt-Shin.

Table 5 shows the results of the statistical model. For the mean rate (parameter *µ*), for every 1% of illiteracy rate, there is a relative increase in the mean incidence rate of severe COVID-19 in pregnant women, which is 5.1%. For the parameter that measures the probability of a municipality having a zero rate (parameter ν), the chance of a municipality having a zero rate increases by 2.7% for every 1% of the proportion of FHS coverage, by 19.3% for every 0.01 of M-HDI education, and by 21.3% for every 0.01 of M-HDI longevity. Population density reduces the chance by 4.5% for every 10 inhabitants/km².

**Table 5:** Estimated coefficients of the zero-adjusted Gamma (ZAGA) statistical model.

Coefficient	Estimate	Standard error	p-value
Parameter *µ*			
Intercept	-3.49	2.52	0.17
M-HDI	5.21	3.28	0.11
Illiteracy rate (15 years and older)	4.96	2.17	0.02
Parameter *σ*			
Intercept	-0.56	0.07	0.00
Parameter *ν*			
Intercept	-0.83	4.47	0.85
Proportion of FHS coverage	2.65	1.44	0.07
M-HDI	-41.18	17.88	0.02
M-HDI education	17.68	9.70	0.07
M-HDI longevity	19.32	8.60	0.03
Population density	0.00	0.00	0.03

FHS: Family Health Strategy; M-HDI: Municipal Human Development Index.

## Discussion

The results showed an association of social vulnerability indicators with severe COVID-19 in pregnant women. With the ZAGA regression method, some indicators showed an influence on the incidence rate of cases or the probability of the incidence rate being zero, namely: M-HDI, illiteracy rate, proportion of FHS coverage, M-HDI education, M-HDI longevity, and population density.

According to studies with the obstetric population during the COVID-19 pandemic, the unfavorable outcomes of pregnant women are not exclusively related to anatomical and physiological factors ^4^ ^,^ ^16^. Social vulnerability indicators also play an important role in the risk of infection. The study by Takemoto et al. ^19^ conducted in Brazil with 978 pregnant women identified that more than half of this group died from COVID-19 and they had no comorbidities or risk factors. It seems to indicate that apparently young and healthy women died due to complications from COVID-19 during pregnancy or shortly after birth, which suggests that inequality is a key element when managing the pandemic, clearly influencing the way it affects the population and, in this case, pregnant women.

The study findings showed that the higher the illiteracy rate in a municipality, the higher the estimated average incidence rate of severe COVID-19 in pregnant women, and the higher the M-HDI Education, the greater the chance of the municipality having a zero incidence rate, which reinforces the understanding that social vulnerability is associated with the possibility of infection and its unfavorable outcomes. A study ^20^ found that patients with no education have COVID-19 incidence rates three times higher (71.3%) than those with higher education (22.5%).

This is because “...*prevention or mitigation policies were not universally applied; on the contrary, they were selective*” ^21^ (p. 27). The privilege of quarantine is not applicable to everyone; in this sense, it is discriminatory, more difficult for some social groups than for others. For those with low income and education, quarantine is particularly unviable. Also, for a large group of caregivers, housemaids, nannies, and Uber drivers whose mission was to make quarantine possible for the entire population, social isolation is not an option. These groups have something in common: “...*they suffer from a special vulnerability that precedes quarantine and becomes worse with it*” ^21^ (p. 15).

Siqueira et al. ^4^ conducted a population-based ecological study to assess the relationship between COVID-19 cases/deaths and socioeconomic variables in the obstetric population in Brazil. They found that municipalities with a high degree of socioeconomic inequality had higher maternal mortality rates than those with better social and infrastructure indicators. The association of social vulnerability with the incidence of severe COVID-19 in pregnant women indicates that socioeconomic inequalities may aggravate in places with structural problems, such as lack of basic sanitation, tap water, and adequate garbage collection, as well as illiteracy and poverty ^22^.

The unequal characteristics of the distribution of SARS-CoV-2 in Pernambuco highlighted this structure of discrepant exposure to risk. Ayres et al. ^23^ attribute the concept of vulnerability to issues that ensure the citizenship of the most politically fragile populations. They understand that vulnerability is associated with three components: individual, social, and programmatic aspects, with social aspects related to the ability to receive information and socially and politically influence free expression, safety, and protection of the individual.

A study suggested that COVID-19 is actually a syndemic and not a pandemic, and the conceptual model of the syndemic explains the spread and consequences of the disease in populations. This is because, according to the theory, the social, economic, and environmental characteristics that determine the living conditions of populations intensify the interaction between coexisting diseases and the excessive burden of the outcomes. It is exactly based on this understanding that the study highlights that the most important issue when considering COVID-19 as a syndemic is the recognition of its social origins ^24^.

In this study, the chance of a municipality having a zero incidence rate of cases of pregnant women with COVID-19 increases with greater coverage of the FHS. The FHS is a strategy used in Brazil that is part of the primary health care (PHC) system, the first level of health care, and is characterized by a set of comprehensive health actions at the individual and collective levels ^15^. In this sense, strong PHC can significantly contribute to handling public emergency situations, which can be observed in the efficiency of PHC in providing health care to the population, with very satisfactory results against maternal and infant mortality, among others, due to its capillarity and knowledge of the territory, which strengthens the bond between the health team and the community, contributing to care comprehensiveness ^25^.

Responsibility for the territory and the people who live there and the possibility of conducting a community-based surveillance are certainly elements that enhance the role of primary care in controlling SARS-CoV-2 infection. PHC is described as a relevant tool in the fight against COVID-19 ^26^, from the first symptoms.

Chioro et al. ^27^ report that during the COVID-19 pandemic, when patients needed to use health services, they did so through primary care at basic health units. However, with the pandemic, these health services were overloaded and, in Brazil, the vulnerabilities that already existed in obstetric care, including difficult access to prenatal care and a shortage of professionals to deal with complications during pregnancy, became worse during this period ^28^.

Regions with high income inequalities usually have worse living conditions, inadequate housing, crowds, and difficult access to health services ^4^. Even with the recommendations of the Brazilian Ministry of Health about the importance of obstetric care and the investigation to identify any risk to the health of these women ^29^, these services had limited access. Today, the state of Pernambuco has 2,412 obstetric beds offered by the Brazilian Unified National Health System (SUS, acronym in Portuguese), of which 321 are for high-risk women.

We can say that the development of health actions should take into account the process of social vulnerability involving the illness of the obstetric population due to COVID-19, which can help effectively control the pandemic. With this identification, it is also possible to monitor health inequalities in the territories and understand their causes, providing an understanding of the impact of social programs on reducing inequalities ^30^.

One of the methodological limitations refers to the use of secondary data from health information systems, which may have led to underreporting, in addition to providing incomplete data. It should be noted that SIVEP-Gripe is not a uniform system, especially in terms of private health units. In addition, indicators calculated from data of the 2010 *Brazilian Demographic Census* were used. And under no circumstances the analysis conducted in this study allows any causal conclusion. To do so, it would be necessary to establish much stronger epidemiological premises (and justify them) and use more appropriate statistical models to make causal inferences.

## Conclusion

This study highlights the impact of social vulnerability indicators on the incidence of severe cases of COVID-19 in pregnant women, pointing to a path of influence of some aspects related to social and demographic characteristics on the behavior of the pandemic. Our study found that municipalities with higher illiteracy rates and smaller coverage of the FHS had a higher risk of incidence and a lower chance of having a zero rate. The pandemic context, together with social determinants, contributes to exposure and vulnerability in different social environments, establishing a dynamic relationship between individuals, society, and the health-disease process. The results of this study can support the planning of actions aiming to control severe COVID-19 in pregnant women, taking into account social vulnerability as a basis for implementing priority strategies.
