# Supplementary material for: Social vulnerability and severe COVID-19 in pregnant women: an ecological study in Pernambuco State, Brazil, 2020-2021
Source: Cad Saude Publica. 2025 Mar 31;41(2):e00175623. doi: 10.1590/0102-311XEN175623 (PMC11960752; doi:10.1590/0102-311XEN175623)
Supplement: Translation

# Translation: Vulnerabilidade social e COVID-19 grave em gestantes: um estudo ecológico em Pernambuco, Brasil, 2020-2021

**DOI:** 10.1590/0102-311XPT175623

**Authors:** Hingrid Wandille Barros da Silva Sá, Mirella Bezerra Rodrigues Vilela, Carlos Fabrício Assunção da Silva, Gabriella Morais Duarte Miranda, Heitor Victor Veiga da Costa, Cristine Vieira do Bonfim

**Affiliations:** 1 Universidade Federal de Pernambuco, Recife, Brasil.; 2 Instituto Aggeu Magalhães, Fundação Oswaldo Cruz, Recife, Brasil.; 3 Fundação Joaquim Nabuco, Recife, Brasil.

**Keywords:** Gestantes, COVID-19, Vulnerabilidade Social

## Abstract

Analisou-se a associação dos indicadores de vulnerabilidade social com a taxa de incidência de casos graves de COVID-19 em gestantes em Pernambuco, Brasil, entre 2020 e 2021. Este é um estudo ecológico com os casos graves de gestantes com COVID-19, notificados no Sistema de Informação de Vigilância Epidemiológica da Gripe. Para a associação entre a vulnerabilidade social e esses casos, aplicou-se o modelo de regressão gama ajustado de zeros (ZAGA), devido ao grande número de zeros na variável resposta. Utilizou-se variáveis disponíveis no censo demográfico, representando condições socioambientais, características domiciliares e de serviços urbanos. Foram registrados 475 casos graves de COVID-19 em gestantes, com taxa de incidência de 1,40 por 1.000 nascidos vivos. A modelagem com o ZAGA mostrou que a média da taxa de incidência de casos é afetada pela taxa de analfabetismo, sendo que a cada 1%, a média aumenta relativamente 5,1% (p = 0,024). O modelo ZAGA também estima a chance de um município ter taxa zero, sendo que esses valores aumentam em: 2,7% a cada 1% de proporção da cobertura da Estratégia Saúde da Família, em 19,3% a cada 0,01 ponto de Índice de Desenvolvimento Humano Municipal (IDH-M) educação e 21,3% a cada 0,01 ponto de IDH-M longevidade. Quando o IDH-M aumenta, a chance de o município ter taxa zero diminui em 33,8% a cada 0,01 ponto. A densidade demográfica diminui a chance em 4,5% a cada 10 habitantes/km^2^. O estudo evidenciou a influência dos indicadores de vulnerabilidade social sobre a incidência de casos de COVID-19 grave em gestantes, em que alguns aspectos referentes às características sociais e demográficas estão relacionados.

## Introdução

A pandemia pela COVID-19 alertou todo o mundo e suscitou debates acerca das medidas de enfrentamento da doença. Essas estratégias estão conectadas com as evidências de que alguns grupos correm maior risco de ter complicações pela COVID-19 ^1^. Entre elas, as alterações fisiológicas do período gestacional predispõem grávidas a formas mais graves da infecção ^2^ ^,^ ^3^, sendo consideradas um grupo de risco e prioritário para assistência e testagem ^2^.

O vírus da COVID-19 se espalhou rapidamente pelo mundo, provocando problemas sociais, econômicos e de saúde em diversos países ^4^. Um estudo realizado no Brasil ^5^ avaliou a relação entre as taxas de incidência, mortalidade e letalidade da COVID-19 e indicadores sociais de desenvolvimento humano e vulnerabilidade social. Segundo a pesquisa, especialmente nos países de baixa e média renda, há preocupação com os efeitos dessa pandemia nas populações mais empobrecidas pela dificuldade de adoção de medidas preventivas. Esses grupos estão expostos a um contexto de vulnerabilidade que aumenta o risco de contaminação e, caso ocorra a infecção, têm acesso limitado aos serviços de saúde.

Assim como em outros agravos de saúde, a vulnerabilidade social pode influenciar o risco de infecção, morbidade e mortalidade associado à COVID-19 ^1^. A vulnerabilidade social consiste em uma definição multidimensional formada por indivíduos ou grupos em situação de fragilidade, seja por fatores biológicos, epidemiológicos, sociais e/ou culturais, que os expõe a riscos e a níveis significativos de desagregação social ^6^.

Diversas pesquisas científicas evidenciaram a existência de desigualdades sociais decorrentes da pandemia pela COVID-19 ^7^ ^,^ ^8^. Esses estudos revelaram desigualdades raciais e étnicas ^9^ ^,^ ^10^, socioeconômicas ^11^ e territoriais na mortalidade associada à doença ^12^. Juntamente com as desigualdades sociais pré-existentes, a pandemia enfatizou ainda mais a questão global das iniquidades sociais em saúde ^13^.

A vulnerabilidade afeta principalmente as pessoas em situação socioeconômica menos favorável, com acesso limitado aos cuidados de saúde e serviços de qualidade insuficiente ^14^. Analisar a associação da vulnerabilidade social com a COVID-19 grave em gestantes pode oferecer suporte à implementação de medidas de controle da propagação do vírus e ao planejamento para a alocação de recursos. Com base nessa perspectiva, o estudo teve como objetivo analisar a associação dos indicadores de vulnerabilidade social com a taxa de incidência de casos graves de COVID-19 em gestantes em Pernambuco, Brasil, entre 2020 e 2021.

## Materiais e métodos

### Área de estudo

Adotou-se uma abordagem ecológica, tendo como unidade de análise os municípios do Estado de Pernambuco. Localizado na Região Nordeste do Brasil, é composto por 184 municípios, além do arquipélago de Fernando de Noronha. De acordo com o *Censo Demográfico* de 2022, a população total do estado era de 9.058.931 habitantes.

### Fonte de dados

Foram incluídos no estudo todos os casos confirmados de gestantes com COVID-19 e manifestações clínicas classificadas pelo Ministério da Saúde como graves, notificados no período de março de 2020 a dezembro de 2021, em Pernambuco. Os casos graves foram extraídos do Sistema de Informação de Vigilância Epidemiológica da Gripe (SIVEP-Gripe), um banco de dados nacional criado pelo Ministério da Saúde em 2009 para a vigilância sentinela da síndrome gripal e que incluiu a notificação dos casos de SRAG e óbitos causados pelo SARS-CoV-2. A variável observada no SIVEP-Gripe para a análise espacial foi o município de residência. O e-SUS Notifica foi utilizado para obtenção do número de casos de COVID-19 em gestantes no período estudado.

As variáveis utilizadas para a extração do banco de dados de síndrome respiratória aguda grave (SRAG) hospitalizados foram: data de preenchimento da ficha de notificação (entre março de 2020 e dezembro de 2021); município de notificação (municípios do Estado de Pernambuco); sexo (feminino); gestante (idade gestacional da paciente). Foi considerado como critério de exclusão a idade materna inferior a 11 anos e superior a 60 anos. Considerou-se caso de SRAG a apresentação de dispneia/desconforto respiratório ou pressão persistente no tórax ou a saturação de oxigênio menor que 95% em ar ambiente ou coloração azulada de lábios ou rosto ^15^.

Para análise da associação com a vulnerabilidade social, foram utilizados os dados sociais, demográficos e econômicos extraídos do *Censo Demográfico* de 2010. Para a variável cobertura da Estratégia Saúde da Família (ESF), foram utilizados os dados do ano de 2020 do sistema e-Gestor Atenção Primária à Saúde (https://egestoraps.saude.gov.br/), um espaço desenvolvido pelo Ministério da Saúde para informações e acesso aos sistemas de atenção básica.

Os indicadores utilizados para a análise da vulnerabilidade social foram: Índice de Vulnerabilidade Social (IVS); Índice de Desenvolvimento Humano Municipal (IDH-M); IDH-M baseado na educação (IDH-M educação); IDH-M baseado na longevidade (IDH-M longevidade); IDH-M baseado na renda (IDH-M renda); Índice de Gini; taxa de desemprego; renda *per capita*; taxa de analfabetismo com 15 anos ou mais; proporção da população com esgoto inadequado; proporção da população com domicílio com água encanada; proporção de coleta de lixo adequado; proporção da população com banheiro e água adequados; proporção da população com domicílio com coleta de lixo; proporção da população com domicílio com energia elétrica; taxa de urbanização e densidade demográfica.

Para o cálculo da taxa de incidência de casos graves em gestantes, foi utilizado no denominador, o número de nascidos vivos registrados nos anos de 2020 e 2021 no Sistema de Informações sobre Nascidos Vivos (SINASC).

### Análise de dados

Com o objetivo de determinar a influência dos indicadores de vulnerabilidade social na taxa de incidência de casos graves de COVID-19 em gestantes a cada 1.000 nascidos vivos (variável resposta), foi calculada a taxa para cada município de Pernambuco durante o período de 2020 a 2021 (onde foi utilizada a média entre os dois anos), levando em consideração o município de residência da gestante, utilizando a seguinte equação:

$${Taxa}_{i}=\frac{TotaldecasosgravesdeCOVID-19emgestantes}{Nascidos{vivos}_{i}}1.000$$

onde *i* = município de cálculo.

Em virtude da variável resposta apresentar alta quantidade de valores iguais a zero, houve dificuldade na modelagem por meio de modelos de regressão usuais. Dessa forma, para a etapa de modelagem, foram testados dois modelos probabilísticos ajustados para valores zero. O primeiro foi o modelo de regressão gama ajustado de zeros (*zero adjusted Gamma* - ZAGA), e o segundo foi o normal inversa ajustada de zeros (*zero adjusted inverse Gaussian* - ZAIG), usando a métrica critério de informação de Akaike generalizado (g*eneralized Akaike information criteria* - GAIC). Esses modelos foram adotados devido à variável resposta ser uma taxa que apresenta valores estritamente maiores ou iguais a zero, sendo que esses modelos se encaixam muito bem para a modelagem desse tipo de variável pois seu suporte é nesse intervalo. A definição do modelo se orientou pela obtenção do menor valor de GAIC alcançado [ZAGA (GAIC = 193,1798); ZAIG (GAIC = 199,9904)], sendo, portanto, escolhido o modelo ZAGA. O GAIC nessa etapa foi calculado considerando um ajuste da função densidade de probabilidade dos modelos propostos ao histograma dos dados da variável resposta.

Tais modelos possuem três parâmetros em suas distribuições de probabilidade, que podem ser modelados simultaneamente por meio do *framework* Modelos Aditivos Generalizado para Localização, Escala e Forma - MAGLEF (*Generalized Additive Model for Location, Scale and Shape*) ^16^. O primeiro parâmetro diz respeito a média (*µ*), o segundo, a sua variação (*σ*) e o terceiro estima a probabilidade de um município ter a taxa igual a zero (*ν*). A cada parâmetro, o modelo estatístico foi sujeito ao processo de seleção de variáveis por meio do algoritmo *stepwise*, utilizando GAIC como métrica ^17^.

A forma funcional a ser definida para o modelo ZAGA é dada da seguinte forma:

*Y~ZAGA(μ,σ,ν)*

*log(μ) = β_0+β_1 X_1+β_2 X_2+⋯+β_p X_p*

*log(σ) = α_0+α_1 X_1+α_2 X_2+⋯+α_p X_p*

*logit(ν) = γ_0+γ_1 X_1+γ_2 X_2+⋯+γ_p X_p*

onde *log*(.) é o logaritmo neperiano, $logit(x)=\frac{1}{1+e^{-x}}$ é a função logística e *p =* número de variáveis explicativas.

Logo, cada parâmetro terá uma interpretação diferente. Para os parâmetros que têm função de ligação *log*(.) (por exemplo, *μ* a média), a avaliação da relação de interesse pode ser feita aplicando o *exp*(*β*_*i*_ *u) para o parâmetro *β*_*i*_ referente à variável de interesse, em que a interpretação será que a cada unidade de medida *u* da variável *X*_*i*_ em avaliação, o parâmetro irá aumentar/diminuir em uma escala percentual se comparado a valores defasados de *X*_*i*_ .

Já para o parâmetro que tem função de ligação *logit*(.), segue-se a mesma regra dos demais. Contudo, sua interpretação é a de que a chance de a taxa ter valor zero irá aumentar/diminuir em uma escala percentual se comparado a valores defasados de *X*_*i*_ .

Para a seleção das variáveis a serem incluídas no modelo estatístico, realizou-se uma análise bivariada por meio de dois métodos distintos. O primeiro método considerou apenas os municípios com taxas diferentes de zero (para sua posterior inclusão no método de seleção de variáveis para o parâmetro *μ*), enquanto o segundo método investigou a probabilidade de um município apresentar taxa zero, considerando todos os municípios (para sua posterior inclusão no método de seleção de variáveis para o parâmetro *ν*). O coeficiente de correlação de Spearman foi empregado no primeiro método, enquanto o teste de Mann-Whitney foi utilizado no segundo. Todas as variáveis que apresentaram valor de p < 0,20 na análise bivariada foram incluídas ao processo de seleção de variáveis via *stepwise*. O valor de p empregado de 0,20 foi adotado para ser mais permissivo no momento de testar a inclusão de variáveis ao modelo estatístico. Algo a ser mencionado é que a modelagem poderia ter sido feita por meio do uso de distribuições discretas para modelar contagens, como a Poisson ou a binomial negativa, fazendo uso da população em risco como *offset*. Entretanto, esses modelos são muito mais complexos computacionalmente de serem estimados (em relação a modelos que utilizam distribuições contínuas), o que dificulta o seu uso.

A correlação de Spearman é uma medida que mede a força da relação entre duas variáveis de forma não linear (relações monotônicas não crescentes/decrescentes). Já o teste de Mann-Whitney serve para comparar, de forma não paramétrica, a diferença entre a distribuição de duas populações, que neste caso, serão os municípios com e sem taxas de casos graves iguais a zero.

Quanto a etapa de seleção de variáveis, é importante deixar claro que a parte em que se calculam as correlações é uma etapa de filtragem para o algoritmo de seleção de variáveis *stepwise*. Sendo assim, não estamos selecionando as variáveis baseadas nas correlações, e sim através do algoritmo de seleção de variáveis, o qual é muito mais robusto e criterioso.

Tratando-se da correlação entre o IDH educação e a taxa de analfabetismo, de fato elas são correlacionadas. Contudo, elas estão alocadas em parâmetros diferentes da distribuição, e a análise do *worm plot* não apresentou problemas, o que indica que as duas variáveis estarem no modelo não está afetando nem a estimativa de seus parâmetros nem a qualidade do ajuste.

Para garantir a qualidade dos achados da análise de regressão, a análise de resíduos foi aplicada, na qual foram feitas as seguintes verificações: normalidade dos resíduos por meio dos testes de Shapiro-Wilk, Jarque-Bera, Kolmogorov-Smirnov, Anderson-Darlin, Cramer-von Mises, Lilliefors, Pearson e Shapiro-Francia; estacionaridade dos resíduos utilizando o teste aumentado de Dickey-Fuller, Philips-Perron e KPSS (Kwiatkowski-Phillips-Schmidt-Shin; patamar e tendência); autocorrelação dos resíduos por meio dos testes de Box-Pierce e Ljung-Box. Também foram avaliados gráficos entre os resíduos e os valores valores ajustados e o tempo como também um gráfico de densidade para os resíduos e um *qq-plot* considerando uma distribuição normal padrão.

O *framework* utilizado para estimação dos modelos (GAMLSS − https://www.gamlss.com/) possui uma representação gráfica para os resíduos chamada worm plot, a qual é útil para identificar possíveis fatores para o mal ajustamento de um modelo. Esse gráfico também foi posto, finalizando a análise dos resíduos.

Todos os cálculos referentes à regressão foram realizados via linguagem R versão 4.0.1 (http://www.r-project.org) com auxílio do pacote GAMLSS versão 5.4-3 ^18^. O nível de significância adotado foi de 5% (para interpretação de significância estatística dos parâmetros do modelo após a seleção de variáveis).

O projeto de pesquisa contou com a anuência da Secretaria Estadual de Saúde de Pernambuco para a cessão do banco de dados e obteve a aprovação do Comitê de Ética em Pesquisa da Universidade Federal de Pernambuco (CAAE: 60548022.1.0000.5208; parecer nº 5.544.400).

## Resultados

Foram registrados pelo e-SUS Notifica 4.121 casos de COVID-19 em gestantes no período estudado, sendo 475 (11,5%) notificados como COVID-19 grave no SIVEP-Gripe. A Tabela 1 apresenta as estatísticas descritivas relacionadas aos indicadores de vulnerabilidade social e a taxa de incidência de casos graves de COVID-19 em gestantes. A taxa média (± desvio padrão − DP) considerando todos os municípios foi de 1,40 caso por mil nascidos vivos (± 1,78). Nota-se que o DP é muito maior do que a média por conta do número de municípios com taxa zero, o que corresponde a 46,2% (85) dos municípios pernambucanos.

**Tabela 1:** Estatística descritiva dos indicadores de vulnerabilidade social dos municípios de Pernambuco, Brasil, 2020 e 2021.

Variável	Mínimo	1º quartil	Mediana	Média	3º quartil	Máximo	DP	Coeficiente de variação
Taxa de gestantes com COVID-19 de forma grave *	0,00	0,00	0,84	1,41	2,23	8,98	1,78	1,27
IDH-M	0,49	0,57	0,59	0,60	0,61	0,77	0,05	0,08
IVS	0,31	0,43	0,48	0,47	0,51	0,66	0,07	0,14
Índice de Gini	0,42	0,49	0,52	0,52	0,55	0,68	0,05	0,09
Taxa de desemprego	0,00	0,19	0,30	0,35	0,49	1,00	0,21	0,61
IDH-M educação	0,35	0,44	0,49	0,49	0,52	0,70	0,07	0,13
IDH-M longevidade	0,68	0,73	0,76	0,76	0,78	0,84	0,03	0,05
IDH-M renda	0,48	0,54	0,57	0,57	0,60	0,80	0,04	0,08
Renda *per capita*	155,49	234,88	271,18	295,96	329,66	1.144,26	104,49	0,35
Taxa de analfabetismo (15 anos ou mais)	0,06	0,23	0,27	0,27	0,32	0,43	0,07	0,26
Proporção de domicílios com esgoto inadequado	0,00	0,11	0,23	0,25	0,35	0,75	0,16	0,62
Proporção da população domiciliar com água encanada	0,00	0,58	0,74	0,71	0,85	1,00	0,19	0,26
Proporção da população domiciliar com banheiro e água adequados	0,00	0,45	0,62	0,60	0,79	1,00	0,23	0,38
Proporção da população domiciliar com coleta de lixo adequada	0,00	0,69	0,84	0,78	0,92	1,00	0,19	0,24
Proporção da população domiciliar com energia elétrica	0,00	0,85	0,91	0,86	0,95	1,00	0,16	0,19
Taxa de urbanização	0,12	0,46	0,62	0,62	0,77	1,00	0,20	0,33
Densidade demográfica	7,79	39,82	87,24	247,04	154,06	9.068,36	905,99	3,67
Proporção da cobertura da ESF	0,00	0,93	1,00	0,93	1,00	1,00	0,14	0,15

DP: desvio padrão; ESF: Estratégia Saúde da Família; IDH-M: Índice de Desenvolvimento Humano Municipal; IVS: Índice de Vulnerabilidade Social.

* Para o cálculo da referida taxa, observou-se um total de 4.121 casos de COVID-19 registrados no e-SUS notifica, dentre os quais 475 eram de gestantes com COVID-19 de forma grave.

A Tabela 2 apresenta a correlação de Spearman entre as variáveis relacionadas aos indicadores de vulnerabilidade social e a taxa de incidência de casos graves de COVID-19 em mulheres grávidas. As variáveis IDH-M, índice de Gini, IDH-M educação, taxa de analfabetismo, proporção da população com água encanada e a proporção da cobertura da ESF tiveram destaque, com valor de p ≤ 0,20.

**Tabela 2:** Correlação de Spearman entre as variáveis explicativas e a variável resposta da taxa de incidência de casos graves de COVID-19 em gestantes, computadas sem a presença de municípios com taxa igual a zero. Pernambuco, Brasil, 2020 e 2021.

Variável	Correlação	Valor de p
IDH-M	-0,146	0,150
IVS	0,037	0,713
Índice de Gini	-0,226	0,025
Taxa de desemprego	-0,121	0,235
IDH-M educação	-0,133	0,188
IDH-M longevidade	-0,087	0,390
IDH-M renda	-0,129	0,202
Renda *per capita*	-0,127	0,211
Taxa de analfabetismo (15 anos ou mais)	0,161	0,111
Proporção da população com esgoto inadequado	0,116	0,254
Proporção da população domiciliar com água encanada	-0,136	0,180
Proporção da população domiciliar com banheiro e água adequados	-0,106	0,298
Proporção da população domiciliar com coleta de lixo adequada	-0,002	0,984
Proporção da população domiciliar com energia elétrica	0,099	0,330
Taxa de urbanização	-0,121	0,235
Densidade demográfica	-0,019	0,855
Proporção da cobertura da ESF	0,220	0,028

ESF: Estratégia Saúde da Família; IDH-M: Índice de Desenvolvimento Humano Municipal; IVS: Índice de Vulnerabilidade Social.

Nota: valores em negrito, testes significativos a nível de 0,20.

Tratando-se da associação entre as variáveis relacionadas aos indicadores de vulnerabilidade social e o fato de um município ter taxa zero ou não (Tabela 3), tem-se que o IVS, índice de Gini e a proporção da população domiciliar com coleta de lixo adequada não apresentaram significância estatística. As demais variáveis estão aptas a entrarem na análise multivariada, sendo que os municípios que tiveram taxa zero tendem a ter menores valores centrais (mediana/média) para IDH-M, taxa de desemprego, IDH-M educação, IDH-M longevidade, IDH-M renda, renda *per capita*, proporção da população domiciliar com água encanada, proporção da população domiciliar com banheiro e água adequados, proporção da população domiciliar com energia elétrica e densidade demográfica.

**Tabela 3:** Estatística descritiva dos indicadores de vulnerabilidade social para os municípios estratificados de acordo com a taxa zero de casos graves de gestantes com COVID-19. Pernambuco, Brasil, 2020-2021.

Variável	Municípios com taxa igual a zero	Mínimo	1º quartil	Mediana	Média	3º quartil	Máximo	DP	Coeficiente variação	Valor de p *
IDH-M	Não	0,52	0,57	0,60	0,61	0,64	0,77	0,05	0,09	0,00
	Sim	0,49	0,57	0,59	0,58	0,60	0,67	0,03	0,06	
IVS	Não	0,31	0,42	0,47	0,46	0,51	0,66	0,07	0,16	0,08
	Sim	0,36	0,45	0,49	0,48	0,51	0,62	0,06	0,12	
Índice de Gini	Não	0,43	0,49	0,52	0,52	0,55	0,68	0,05	0,09	0,97
	Sim	0,42	0,49	0,52	0,52	0,55	0,63	0,04	0,08	
Taxa de desemprego	Não	0,00	0,24	0,36	0,39	0,53	1,00	0,21	0,55	0,00
	Sim	0,01	0,16	0,25	0,31	0,41	0,87	0,20	0,66	
IDH-M educação	Não	0,37	0,45	0,50	0,50	0,55	0,70	0,07	0,14	0,02
	Sim	0,35	0,44	0,48	0,47	0,52	0,58	0,05	0,11	
IDH-M longevidade	Não	0,68	0,73	0,76	0,76	0,78	0,84	0,04	0,05	0,06
	Sim	0,68	0,73	0,75	0,75	0,77	0,81	0,03	0,04	
IDH-M renda	Não	0,50	0,55	0,58	0,59	0,61	0,80	0,05	0,09	0,00
	Sim	0,48	0,54	0,56	0,56	0,58	0,65	0,03	0,05	
Renda *per capita*	Não	175,50	249,20	292,7	323,90	363,60	1.144,30	128,10	0,40	0,00
	Sim	155,50	232,30	256,3	263,40	297,00	441,80	51,40	0,20	
Taxa de analfabetismo (15 anos ou mais)	Não	0,06	0,21	0,26	0,25	0,32	0,40	0,08	0,31	0,00
	Sim	0,15	0,25	0,29	0,29	0,33	0,43	0,06	0,19	
Proporção da população com esgoto inadequado	Não	0,00	0,09	0,18	0,21	0,31	0,71	0,16	0,73	0,00
	Sim	0,05	0,19	0,30	0,30	0,40	0,75	0,14	0,49	
Proporção da população domiciliar com água encanada	Não	0,22	0,65	0,81	0,75	0,90	1,00	0,18	0,24	0,00
	Sim	0,00	0,52	0,67	0,65	0,79	0,98	0,18	0,27	
Proporção da população domiciliar com banheiro e água adequados	Não	0,07	0,51	0,72	0,67	0,83	1,00	0,23	0,34	0,00
	Sim	0,00	0,40	0,52	0,53	0,68	0,99	0,21	0,40	
Proporção da população domiciliar com coleta de lixo adequada	Não	0,00	0,69	0,84	0,78	0,92	1,00	0,19	0,25	0,77
	Sim	0,03	0,69	0,85	0,79	0,91	1,00	0,19	0,24	
Proporção da população domiciliar com energia elétrica	Não	0,00	0,89	0,93	0,89	0,97	1,00	0,16	0,18	0,00
	Sim	0,12	0,76	0,88	0,83	0,93	0,99	0,16	0,19	
Taxa de urbanização	Não	0,06	0,21	0,26	0,25	0,32	0,40	0,08	0,31	0,00
	Sim	0,15	0,25	0,29	0,29	0,33	0,43	0,06	0,19	
Densidade demográfica	Não	8,00	58,10	118,80	390,00	196,40	9.068,40	1.218,10	3,10	0,00
	Sim	7,80	27,50	58,30	80,60	104,90	328,20	71,40	0,90	
Proporção da cobertura da ESF	Não	0,45	0,86	1,00	0,91	1,00	1,00	0,14	0,16	0,00
	Sim	0,00	1,00	1,00	0,97	1,00	1,00	0,12	0,13	

DP: desvio padrão; ESF: Estratégia Saúde da Família; IDH-M: Índice de Desenvolvimento Humano Municipal; IVS: Índice de Vulnerabilidade Social.

Nota: valores em negrito, testes significativos a nível de 0,05.

* Teste de Mann-Whitney

Já as variáveis taxa de analfabetismo (15 anos ou mais), proporção da população com esgoto inadequado e taxa de urbanização possuem valores centrais superiores para os municípios com taxa zero.

A variável proporção da cobertura da ESF tem mediana igual para ambos os estratos de municípios, contudo, sua média é maior em municípios com taxa zero. Isso indica que abaixo da mediana, a proporção de cobertura dos municípios com taxa zero tende a ser maior em relação aos municípios com taxa diferente de zero.

Antes de apresentar os resultados do modelo estatístico, é apresentada a análise dos resíduos (Tabela 4 e Figuras 1 e 2). Nenhum dos testes aplicados apresentou inconformidades quanto aos resíduos do modelo (normalidade, estacionaridade e autocorrelação). A Figura 1 também não demonstrou nenhuma divergência quanto as suposições de heterocedasticidade e as demais. Por fim, o *worm plot* (Figura 2) destaca todos os pontos dentro das bandas de confiança, o que é um sinal de que não há divergências significativas dos resíduos ao que se pressupõe a eles. Portanto, os resultados do modelo de regressão podem ser considerados confiáveis.

**Tabela 4:** Testes estatísticos realizados para verificar normalidade, estacionaridade e autocorrelação nos resíduos do modelo gama ajustado de zeros (ZAGA).

Teste realizado	Valor de p	Resultado
Shapiro-Wilk	0,51	É normal
Jarque-Bera	0,98	É normal
Kolmogorov-Smirnov	0,82	É normal
Anderson-Darlin	0,45	É normal
Cramer-von Mises	0,41	É normal
Lilliefors	0,37	É normal
Pearson	0,65	É normal
Shapiro-Francia	0,59	É normal
Augmented Dickey-Fuller	0,01	É estacionário
Philips-Perron	0,01	É estacionário
KPSS for Level	0,10	É estacionário
KPSS for Trend	0,10	É estacionário
Box-Pierce	1,00	Sem autocorrelação
Ljung-Box	1,00	Sem autocorrelação

KPSS: Kwiatkowski-Phillips-Schmidt-Shin.

**Figura 1:** Gráficos referentes a análise de resíduos.

**Figura 2:** *Worm plot* para os resíduos do modelo.

Os resultados do modelo estatístico estão na Tabela 5. Para a média da taxa (parâmetro *µ*), tem-se que a cada 1% de taxa de analfabetismo, há um aumento relativo na taxa média de incidência de casos graves de COVID-19 em gestantes, que é de 5,1%. Já para o parâmetro que mede a probabilidade de um município ter taxa zero (parâmetro *ν*), tem-se que a chance de um município ter taxa zero aumenta em 2,7% a cada 1% de proporção da cobertura da ESF, em 19,3% a cada 0,01 ponto de IDH-M educação e 21,3% a cada 0,01 ponto de IDH-M longevidade. A densidade demográfica diminui a chance em 4,5% a cada 10 habitantes/km^2^.

**Tabela 5:** Estimativas dos coeficientes do modelo estatístico gama ajustado de zeros (ZAGA).

Coeficiente	Estimativa	Erro padrão	Valor de p
Parâmetro *µ*			
Intercepto	-3,49	2,52	0,17
IDH-M	5,21	3,28	0,11
Taxa de analfabetismo (15 anos ou mais)	4,96	2,17	0,02
Parâmetro *σ*			
Intercepto	-0,56	0,07	0,00
Parâmetro *ν*			
Intercepto	-0,83	4,47	0,85
Proporção da cobertura ESF	2,65	1,44	0,07
IDH-M	-41,18	17,88	0,02
IDH-M educação	17,68	9,70	0,07
IDH-M longevidade	19,32	8,60	0,03
Densidade demográfica	0,00	0,00	0,03

ESF: Estratégia Saúde da Família; IDH-M: Índice de Desenvolvimento Humano Municipal.

## Discussão

Os resultados demonstraram a associação dos indicadores de vulnerabilidade social com a COVID-19 grave em gestantes. Com a aplicação do método de regressão ZAGA, foi detectado que alguns indicadores podem influenciar a taxa de incidência de casos ou a probabilidade da taxa de incidência ser zero. São eles: IDH-M; taxa de analfabetismo; proporção da cobertura da ESF; IDH-M educação; IDH-M longevidade; e densidade demográfica.

Estudos com a população obstétrica durante a pandemia pela COVID-19 têm mostrado que os desfechos desfavoráveis das gestantes não estão exclusivamente relacionados a fatores anatômicos e fisiológicos ^4^ ^,^ ^16^, com os indicadores de vulnerabilidade social também desempenhando um papel importante no risco de infecção. O estudo de Takemoto et al. ^19^ realizado no Brasil com 978 gestantes identificou que mais da metade desse grupo morreu por COVID-19, e elas não tinham comorbidade ou fatores de risco registrados. Isso parece apontar que mulheres aparentemente jovens e saudáveis morreram devido às complicações da COVID-19 durante a gravidez ou logo após o nascimento, o que sugere que a iniquidade é elemento-chave a ser considerado no enfrentamento da pandemia e que interfere claramente na forma com que acomete a população e, nesse caso, as gestantes.

Os achados do estudo mostraram que, quanto maior a taxa de analfabetismo do município, maior será a estimativa da taxa média de incidência de casos de COVID-19 grave em gestantes, e quanto maior o IDH-M educação, mais chance de o município ter taxa zero de incidência de casos, o que fortalece a compreensão de que a vulnerabilidade social tem associação com a possibilidade de ocorrência da infecção e de seus desfechos desfavoráveis. Um estudo ^20^ identificou que os pacientes sem escolaridade têm taxas de incidência de COVID-19 três vezes superiores (71,3%) aos que têm Nível Superior (22,5%).

Isso ocorre porque “...*as políticas de prevenção ou de contenção não foram de aplicação universal, foram pelo contrário, seletivas*” ^21^ (p. 27). O privilégio da quarentena não se estende a todo(as); nesse sentido, ela é discriminatória, mais difícil para uns grupos sociais do que para outros. Para aqueles de baixa renda e escolaridade, é particularmente impraticável. Ademais, para um vasto grupo de cuidadores, empregadas domésticas, babás, motoristas de Uber, cuja missão foi tornar possível a quarentena ao conjunto da população, o isolamento social não é uma opção. Esses grupos têm algo em comum: “...*padecerem de uma especial vulnerabilidade que precede a quarentena e se agrava com ela*” ^21^ (p. 15).

Siqueira et al. ^4^ realizaram um estudo ecológico de base populacional para verificar a relação entre os casos/óbitos de COVID-19 e as variáveis socioeconômicas na população obstétrica no Brasil, em que municípios com alto grau de dissimilaridade socioeconômica apresentaram maiores razões de mortalidade materna do que áreas com melhores indicadores sociais e de infraestrutura. A associação da vulnerabilidade social com a incidência por COVID-19 grave em gestantes indica que as desigualdades socioeconômicas podem se agravar em locais com problemas estruturais, como falta de saneamento básico, água encanada, coleta adequada de lixo, e com analfabetismo e pobreza ^22^.

As características desiguais da distribuição da SARS-CoV-2 em Pernambuco evidenciaram essa estrutura de disparidade na exposição ao risco. Ayres et al. ^23^ atribuem o conceito de vulnerabilidade às questões que asseguram a cidadania das populações mais frágeis politicamente. Eles compreendem que a vulnerabilidade está associada a três componentes, o individual, o social e o programático, sendo o social relacionado à capacidade de receber informações e influenciar social e politicamente na livre expressão, na segurança e na proteção do indivíduo.

Um estudo sugeriu que a COVID-19 na verdade é uma sindemia, e não uma pandemia. E que por isso, o modelo conceitual da sindemia é aquele que melhor explica a disseminação e as consequências da doença nas populações. Isso porque, de acordo com a teoria, as características sociais, econômicas e ambientais determinantes das condições de vida das populações intensificam a interação entre as doenças coexistentes e a carga excessiva das consequências resultantes. É exatamente a partir dessa compreensão que o estudo destaca que a questão mais importante ao se considerar a COVID-19 como uma sindemia é o reconhecimento de suas origens sociais ^24^.

Nesse estudo, a chance de um município ter taxa zero de incidência de casos de gestantes com COVID-19 aumenta quanto maior for a cobertura da ESF. Ela é uma estratégia utilizada no Brasil que faz parte da atenção primária à saúde (APS), primeiro nível de atenção em saúde, e se caracteriza por um conjunto de ações integrais de saúde no âmbito individual e coletivo ^15^. Nesse sentido, uma APS forte tem capacidade de contribuir de modo importante no enfrentamento de situações de emergências públicas, e isso pode ser evidenciado na eficiência da APS no cuidado em saúde da população, que vem apresentando resultados bastante satisfatórios no combate à mortalidade materna e infantil (e de outras questões) decorrente de sua capilaridade e do conhecimento do território, que fortalece o vínculo entre a equipe de saúde e a comunidade, contribuindo para a integralidade da assistência ^25^.

A responsabilidade pelo território e pelas pessoas que nele habitam e a possibilidade de realização de uma vigilância de base comunitária com certeza são elementos que potencializam o papel da atenção primária no controle da infecção por SARS-CoV-2. A APS é descrita como um relevante dispositivo no enfrentamento à COVID-19 ^26^, desde os primeiros sintomas.

Chioro et al. ^27^ referem que durante a pandemia pela COVID-19, quando os pacientes precisaram usar os serviços de saúde, fizeram-no pela atenção primária, por intermédio das unidades básicas de saúde (UBS). Porém, durante a pandemia, houve uma sobrecarga desses serviços de saúde e, no Brasil, as vulnerabilidades que já existiam na assistência obstétrica, incluindo dificuldades de acesso ao pré-natal e restrição de profissionais para lidar com complicações na gravidez, pioraram ^28^.

As áreas com grandes desigualdades de renda geralmente apresentam piores condições de vida, moradia inadequada, aglomeração e uma maior dificuldade de acesso aos serviços de saúde ^4^. Mesmo diante das recomendações do Ministério da Saúde sobre a necessidade de se manter o atendimento à população obstétrica, assim como a investigação que representasse risco para a saúde dessas mulheres ^29^, esses serviços tiveram acesso limitado. O Estado de Pernambuco possui hoje 2.412 leitos de obstetrícia disponíveis para o Sistema Único de Saúde (SUS), sendo que desses, 321 são de alto risco.

É factível interpretar que a elaboração das ações em saúde precisa levar em conta o processo de vulnerabilidade social que envolve o adoecimento da população obstétrica por COVID-19, o que pode auxiliar o controle efetivo da pandemia. Com essa identificação, também é possível monitorar as desigualdades em saúde nos territórios e entender as suas causas, favorecendo a avaliação do impacto dos programas sociais na redução das desigualdades ^30^.

Uma das limitações metodológicas consiste no uso de dados secundários oriundos de sistemas de informação em saúde, o que pode ter implicado em subnotificação, além da incompletude dos dados. Considera-se que o SIVEP-Gripe não é uniforme, especialmente em se tratando de unidades de saúde privadas. Acrescenta-se também o fato de estarem sendo utilizados indicadores calculados a partir de dados do *Censo Demográfico* de 2010. Ainda, sob nenhuma circunstância, a análise realizada neste estudo permite qualquer conclusão causal. Para tanto, seria necessário estabelecer premissas epidemiológicas muito mais fortes (e justificá-las) e fazer uso de modelos estatísticos mais apropriados para realizar inferências causais.

## Conclusão

O presente estudo evidencia o impacto dos indicadores de vulnerabilidade social na incidência de casos graves de COVID-19 em gestantes, apontando um caminho para a influência de alguns aspectos referentes às características sociais e demográficas no comportamento da pandemia. Verificou-se que os municípios com maior taxa de analfabetismo e menor cobertura da ESF tiveram maior risco de incidência de casos e menor chance de ter taxa zero. O contexto pandêmico, juntamente com os determinantes sociais, contribui para a exposição e a vulnerabilidade em diferentes ambientes sociais, estabelecendo uma relação dinâmica entre indivíduos, sociedade e saúde-doença. Os resultados deste estudo podem auxiliar no planejamento de ações direcionadas ao controle da COVID-19 grave em gestantes, levando em consideração a vulnerabilidade social como base para a implementação de estratégias prioritárias.
